# Transcriptome sequencing and phylogenetic analysis of four species of luminescent beetles

**DOI:** 10.1038/s41598-017-01835-9

**Published:** 2017-05-12

**Authors:** Kai Wang, Wei Hong, Hengwu Jiao, Huabin Zhao

**Affiliations:** Department of Ecology and Hubei Key Laboratory of Cell Homeostasis, College of Life Sciences, Wuhan University, Wuhan, 430072 China

## Abstract

The evolution of bioluminescence has prompted scientific attention to illuminate phylogenetic relationships of luminescent beetles. However, genomic resources are virtually lacking in rhagophthalmids (Rhagophthalmidae) and their related firefly beetles lampyrids (Lampyridae). Here, we employed the Illumina Hiseq 2000 platform and sequenced the whole-body transcriptomes of the four luminescent beetles: one rhagophthalmid (*Rhagophthalmus* sp.) and three fireflies (*Asymmetricata circumdata*, *Aquatica ficta*, and *Pyrocoelia pectoralis*). We obtained 55.4, 43.4, 38.6, and 36.7 million clean reads for the four species, respectively. All reads were assembled into contigs from which unigenes were derived. All unigenes were annotated by publicly available databases, and a total of 4325 orthologous genes were identified. Using multiple phylogenetic approaches, our transcriptome data confirmed the distinctiveness of Rhagophthalmidae from Lampyridae, which was also supported by our mitogenome analysis using three newly determined mitogenome sequences and 12 previously published ones. Together, this study is the first report of whole transcriptome sequencing data in Rhagophthalmidae and Lampyridae species, representing a valuable genomic resource for studying the origin and evolution of some remarkable traits in these beetles such as bioluminescence. Moreover, our transcriptome and mitogenome data provide useful phylogenetic information that could be of importance in future studies of phylogenetic inference.

## Introduction

Bioluminescence is among the most spectacular features in living organisms, including numerous species of marine fishes, marine invertebrates, terrestrial invertebrates, fungi, bacteria, and protists^1, 2^. The uses of bioluminescence in nature involve a range of vital functions: camouflage, attraction, defense, warning, communication, mimicry, and illumination^2^. The chemical basis of the natural light-producing molecules has been elucidated in the last century^3^, permitting discoveries of countless valuable applications in biology and medicine using luciferase-based systems^2^. In addition, industrial designers have been ambitious in utilizing the natural light-producing systems in bioluminescent organisms for decoration and street lighting^4^.

Among these bioluminescent organisms, luminescent insects were primarily found in members of the three orders: Collembola (springtails), Diptera (flies), and Coleoptera (beetles)^5^. Within the superfamily Elateroidea of the order Coleoptera, several groups of beetles are able to produce and emit light such as fireflies (Lampyridae), railroad worms (Phengodidae), click beetles (Elateridae), and Rhagophthalmidae^5, 6^. The origin and evolution of bioluminescence in these beetles has prompted a number of studies to illuminate the phylogenetic relationships of these bioluminescent beetles^7–16^. Although both morphological^10–12, 14, 16^ and molecular^7–9, 13, 15, 17^ features were involved in these studies, molecular evidence has been expected to resolve taxonomic status and phylogenetic relationships that have been contentious based on morphological evidence^18^, because the evolutionary history of morphological features is usually complex^18, 19^. Unfortunately, the incongruence of molecular evidence has also been observed in phylogenetic analyses concerning the phylogenetic relationships of these beetles^7–9, 13, 15^, possibly because a single or a few genes lack sufficient phylogenetic signals^20^. For instance, Rhagophthalmidae had previously been assigned to the family Phengodidae inferred from morphological data^11^; other studies based on morphological, embryological and molecular evidence had assigned Rhagophthalmidae to be a subfamily or genus in the family Lampyridae^8, 14, 15, 21^; but more recent evidence from both morphological and molecular data had supported the familial status of Rhagophthalmidae^7, 9, 10, 13, 16, 22–24^, which is distinct from both Lampyridae and Phengodidae. In addition, the taxonomic studies focusing on Lampyridae and Rhagophthalmidae have been scarce, especially for species distributed in China^15, 25, 26^. The scarcity calls for additional data of taxonomic importance. Phylogenomics, i.e. phylogenetic analysis involving genome-scale data, has been believed to outcompete single-gene phylogenetics, which frequently yielded conflicting results caused by stochastic errors from small-scale data sets^20^. However, genomic resources are extremely limited in Rhagophthalmidae and its related beetles, which may prevent their phylogenetic relationships from in-depth investigations.

To provide novel genomic resources from Rhagophthalmidae and its related firefly beetles (Lampyridae), we employed the Illumina Hiseq 2000 platform and sequenced the whole-body transcriptomes of the four beetles: one rhagophthalmid beetle (*Rhagophthalmus* sp.) and three representatives of lampyrid beetles (*Asymmetricata circumdata*, *Aquatica ficta*, and *Pyrocoelia pectoralis*). To validate the phylogenetic inference based on transcriptome data, we employed the traditional Sanger sequencing to obtain two complete mitogenomes (*Aquatica ficta* and *A*. *wuhana*) and one nearly complete mitogenome (*Lamprigera yunnana*); we also identified the 13 protein coding genes (PCGs) in mitogenomes from each of the four whole-body transcriptomes (Supplementary Table S1); and we additionally retrieved 12 published complete mitogenomes from members of Rhagophthalmidae, Lampyridae, Phengodidae, Elateridae, Lycidae, Cantharidae, and Tenebrionidae (see the species names and GenBank accessions in Materials and Methods). Our transcriptome analysis recognized the distinctiveness of Rhagophthalmidae from Lampyridae, which was also supported by our mitogenome analysis.

## Results

### Transcriptome sequencing and assembly

Total RNA was isolated from each of the frozen whole-bodies of beetles. mRNA was purified from the total RNA using oligo-dT attached magnetic beads. Following mRNA fragmentation, cDNA synthesis, end repair, 3’ adenylation, adaptor ligation, and PCR enrichment, four RNA sequencing (RNA-Seq) libraries were constructed using the Illumina TruSeq RNA sample preparation kit. The raw sequence data were filtered by removing adaptors, low-quality reads, and ambiguous reads. We ultimately obtained 55.4, 43.4, 38.6, and 36.7 million clean reads for *Rhagophthalmus* sp., *A*. *circumdata*, *A*. *ficta*, and *P*. *pectoralis*, respectively (Table 1). These clean reads were separately assembled into 44883, 57254, 71424, and 80017 contigs, in which 15418, 24275, 31520, and 31356 unigenes (i.e. unique putative genes) were derived, respectively (Table 1). It appears that the transcriptome data in greater size tend to have less assembled contigs or unigenes. For example, the largest data (*Rhagophthalmus* sp.) contains the least contigs, while the smallest data (*P*.*pectoralis*) consists of the most contigs (Table 1). Details of clean reads, assembled contigs and unigenes of the four transcriptomes generated in this study were given in Table 1. In addition, we compared the 13 PCGs in *A*. *ficta* mitogenome identified from the Illumina-based transcriptome assembly with the same genes obtained from the traditional Sanger sequencing for another individual of the same species, and found that a total of 99.3% sequences from both sequencing approaches are identical. This finding suggests that our transcriptome assemblies are reliable and our sequencing coverages are acceptable.

**Table 1:** Statistics of clean reads, assembled contigs and unigenes of the four transcriptomes generated in this study. Abbreviation: nt, nucleotide(s).

Species	*Rhagophthalmus* sp.	A. *circumdata*	*A*. *ficta*	*P*. *pectoralis*
Clean reads				
Number of reads	55,374,194	43,437,504	38,642,102	36,663,932
Number of bases (nt)	5,496,107,276	3,991,132,686	3,909,904,630	3,532,502,346
Mean length (nt)	99	96	96	96
Contigs				
Number	44,883	57,254	71,424	80,017
Mean length (nt)	1,461	1,087	1,166	1,283
N50 statistics (nt)	2,981	2,016	2,461	2,622
Unigenes				
Number	15,418	24,275	31,520	31,356
Mean length (nt)	1,451	958	838	923
N50 statistics (nt)	2,197	1,556	1,437	1,690

### Functional annotation of unigenes

**Figure 1:**
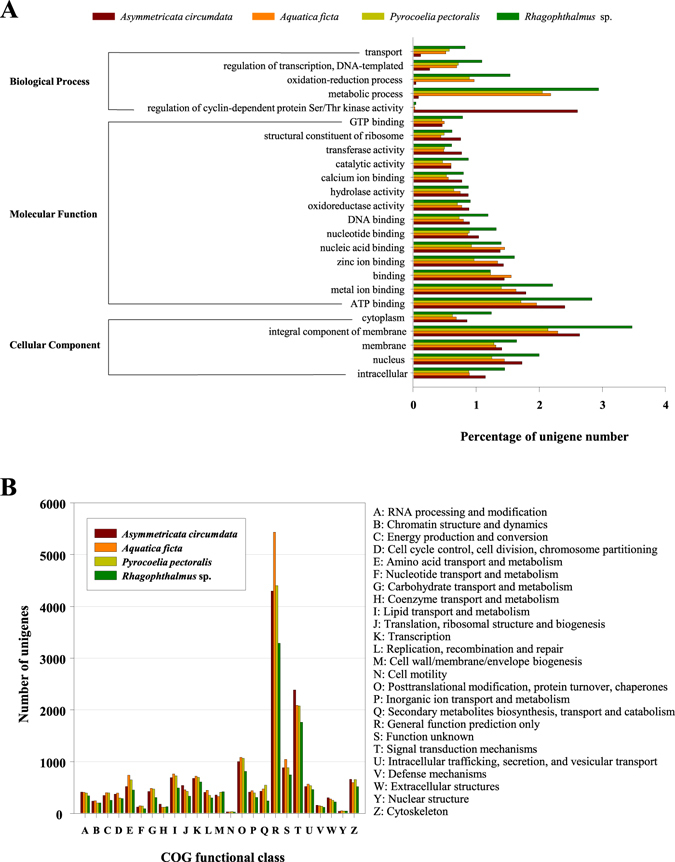
Functional classification of unigenes derived from the four transcriptomes based on the GO (Gene Ontology) annotation (**A**) and the COG (Cluster of Orthologous Groups) annotation (**B**).

A total of 10345 (percentage of all unigenes in a species: 67.10%), 15352 (63.24%), 17480 (55.46%),and 15342 (48.93%) unigenes of *Rhagophthalmus* sp., *A*. *circumdata*, *A*. *ficta*, and *P*. *pectoralis* were respectively annotated by at least one of the publicly available databases: Swiss-prot, NCBI non-redundant protein (NR), Clusters of Orthologous Groups of proteins (COG), and Gene Ontology (GO) (Table 2, Supplementary Dataset S1). We used all unigenes as query and ran the TBLASTX program to search against the Swiss-prot, NR, COG, and GO databases with an E-value of 1.0E-5. For example, we identified 10322 best blast hits in the NR database when annotating the transcriptome of *Rhagophthalmus* sp., comprising 66.95% of all unigenes (Table 2); while only 4845 best hits were detected in the GO database for the same species, covering 31.42% of all unigenes (Table 2). Indeed, the NR database generally annotated the highest percentages of unigenes, whereas the GO database typically annotated the lowest (Table 2). Overall, *Rhagophthalmus* sp. has a higher percentage of unigenes being annotated compared to the other three beetles (Table 2), possibly because a greater size of sequencing data can give a better assembly for the *Rhagophthalmus* beetle (Table 1). In addition, the distributions of both GO terms and COG classes were similar among the four transcriptomes, except *A*. *circumdata* showing a unique expansion in a single GO term (Fig. 1).

**Table 2:** Summary of unigene annotations for the four transcriptomes.

Database	*Rhagophthalmus* sp.	*A. circumdata*	*A. ficta*	*P. pectoralis*
Swiss-prot	8,447 (54.79%)	11,171 (46.02%)	12,042 (38.20%)	11,263 (35.92%)
NR	10,322 (66.95%)	15,315 (63.09%)	17,432 (55.30%)	15,304 (48.81%)
GO	4,845 (31.42%)	6,403 (26.38%)	7,235 (22.95%)	6,428 (20.50%)
COG	9,027 (58.55%)	12,197 (52.25%)	13,357 (42.38%)	12,415 (39.59%)
Total	10,345 (67.10%)	15,352 (63.24%)	17,480 (55.46%)	15,342 (48.93%)

Percentages of all unigenes in a given species were shown in parentheses. Abbreviations: NR, NCBI non-redundant proteins; COG, Clusters of Orthologous Groups of proteins; GO, Gene Ontology.

### Ortholog identification

Genes from the reference genome of the red flour beetle *Tribolium castaneum* were respectively compared with all unigenes from each of the four transcriptome assemblies using the TBLASTX searches^27^. By examining reciprocal (or bi-directional) best blast hits, putative orthologs were identified. If the BLAST score ratio of the second best-hit to the first best-hit is greater than 0.8, we excluded the putative ortholog for further analysis, because the candidate ortholog is possibly a paralog due to the high level of sequence similarity. The resulting putative orthologs range from 6992 to 7612 (Table 2). Each ortholog from the red flour beetle and the four beetles with transcriptome data was aligned by PRANK version 100802^28^, and poorly aligned positions and divergent regions in alignments were filtered by GBLOCKS version 0.91b^29^. After discarding the alignments with an aligned region shorter than 100 nt (nucleotides), we obtained 4325 putative orthologs with high-quality alignments for subsequent analyses.

### Mitogenome sequencing

Using the traditional Sanger sequencing approach, we sequenced two complete mitogenomes (*A*. *ficta* and *A*. *wuhana*) and one nearly complete mitogenome (*L*. *yunnana*) (Table 3). We were unable to sequence the region between *ND2* and *12S rRNA* in *L*. *yunnana* despite multiple attempts (Table 3), possibly because this region contains the A+T-rich region which may pose technical issues in sequencing^30^. All the 13 protein coding genes (PCGs) from each of the three mitogenomes were complete except the *ND2* in *L*. *yunnana* lacking a segment near its 5’ end (Table 3). All the 13 PCGs, 2 rRNAs, 22 transfer RNAs (tRNAs) and 1 A-T-rich region common to the vast majority of animal mitogenomes^31^ were identified in the three newly sequenced mitogenomes, except for the three tRNAs (tRNA^Trp^, tRNA^Cys^, tRNA^Tyr^) and the A-T-rich region in *L*. *yunnana* (Table 3). Arrangements and orientations of all genes in the three mitogenomes are identical to other beetles^25, 32–34^. Similar to other firefly beetles, all the PCGs employed traditional mitochondrial start codons ATN and terminated with TAA, TAG or single T (Table 3).

**Table 3:** Annotations of the three newly sequenced mitochondrial genomes.

Gene	Direction	*Aquatica wuhana*			*Aquatica ficta*			*Lamprigera yunnana*		
		From	To	Start/stop codon	From	To	Start/stop codon	From	To	Start/stop codon
*tRNA* ^*Ile*^	Forward	1	64	ATA/TAA	1	63	ATA/TAG			n.a./TAA
*tRNA* ^*Gln*^	F	62	130		61	129				
*tRNA* ^*Met*^	Reverse	130	195		129	194				
*ND2*	F	196	1209		195	1208		1^*^	878	
*tRNA* ^*Trp*^	F	1211	1275		1210	1274		882	942	
*tRNA* ^*Cys*^	R	1342	1404		1340	1402		1143	1203	
*tRNA* ^*Tyr*^	R	1404	1466		1402	1465		1203	1265	
*COI*	F	1438	3003	ATT/TAA	1458	3002	ATT/TAA	1237	2802	ATT/TAA
*tRNA* ^*Leu*^	F	2999	3062		2988	3061		2798	2861	
*COII*	F	3064	3742	ATG/T+tRNA	3063	3741	ATG/T+tRNA	2835	3540	ATA/T+tRNA
*tRNA* ^*Lys*^	F	3743	3813		3742	3812		3541	3610	
*tRNA* ^*Asp*^	F	3813	3876		3812	3874		3610	3675	
*ATP8*	F	3877	4032	ATT/TAA	3875	4030	ATT/TAA	3685	3828	ATA/TAA
*ATP6*	F	4026	4700	ATG/TAA	4024	4698	ATG/TAA	3822	4485	ATG/T+tRNA
*COIII*	F	4700	5483	ATG/T+tRNA	4698	5481	ATG/T+tRNA	4486	5266	ATG/T+tRNA
*tRNA* ^*Gly*^	F	5484	5546		5482	5545		5267	5328	
*ND3*	F	5547	5900	ATA/TAG	5546	5899	ATT/TAG	5335	5682	ATA/TAG
*tRNA* ^*Ala*^	F	5899	5962		5898	5961		5681	5744	
*tRNA* ^*Arg*^	F	5962	6026		5961	6025		5743	5805	
*tRNA* ^*Asn*^	F	6026	6090		6025	6090		5804	5865	
*tRNA* ^*Ser*^	F	6091	6157		6091	6157		5858	5920	
*tRNA* ^*Glu*^	F	6158	6220		6158	6221		5921	5983	
*tRNA* ^*Phe*^	R	6219	6281		6220	6283		5982	6041	
*ND5*	R	6282	7989	ATT/T+tRNA	6284	7994	ATA/T+tRNA	6039	7749	ATT/T+tRNA
*tRNA* ^*His*^	R	7990	8052		7992	8054		7750	7810	
*ND4*	R	8053	9379	ATG/T+tRNA	8055	9381	ATG/T+tRNA	7810	9135	ATG/TAG
*ND4L*	R	9373	9663	ATG/TAA	9375	9665	ATG/TAA	9129	9416	ATG/TAA
*tRNA* ^*Thr*^	F	9665	9727		9667	9728		9418	9480	
*tRNA* ^*Pro*^	R	9727	9790		9729	9794		9481	9544	
*ND6*	F	9792	10277	ATA/TAA	9796	10284	ATA/TAA	9546	10049	ATT/TAA
*CYTB*	F	10277	11410	ATG/TAG	10284	11417	ATG/TAG	10050	11177	ATG/TAG
*tRNA* ^*Ser*^	F	11409	11474		11416	11481		11176	11240	
*ND1*	R	11479	12437	ATT/TAG	11499	12443	ATT/TAG	11257	12207	ATG/TAG
*tRNA* ^*Leu*^	R	12445	12506		12451	12513		12209	12269	
*16SrRNA*	R	12507	13773		12514	13776		12270	13525	
*tRNA* ^*Val*^	R	13774	13842		13777	13846		13525	13592	
*12SrRNA*	R	13843	14588		13847	14596		13588	14138^*^	
A+T-rich region		14589	16186		14597	16836		n.a.		

Incomplete sequenced region was indicated with an asterisk. Abbreviation: n.a., not available (due to incomplete sequencing).

### Phylogenetic analysis based on nuclear gene sequences

**Figure 2:**
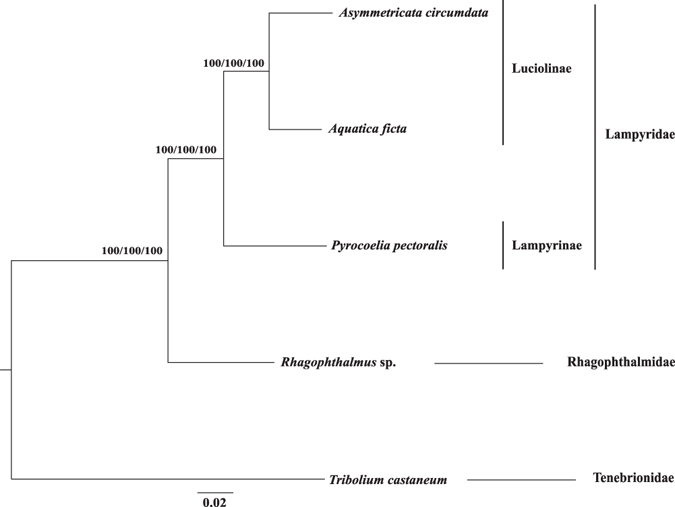
Phylogenetic relationships between Rhagophthalmidae and Lampyridae inferred from the concatenated 4325 nuclear gene markers with the third codon positions removed, *Tribolium castaneum* was chosen to be the outgroup. Numbers at each node are the ML bootstrap values/Bayesian posterior probabilities/MP-EST bootstrap values, shown as percentages.

Phylogenetic analysis was first undertaken with transcriptome-derived nuclear gene sequences. Given that Lampyridae was divided into two monophyletic clades, with one clade consisting of Lampyrinae and the other clade comprising Cyphonocerinae, Ototetrinae, Luciolinae^13^, we selected one species *P*. *pectoralis* (Lampyrinae) from the first clade and two species *A*. *ficta* (Luciolinae) and *A*. *circumdata* (Luciolinae) from the second clade to perform whole-body transcriptome sequencing, in addition to the species *Rhagophthalmus* sp. The resulting transcriptome data were assembled and annotated, and a total of 4325 putative orthologs were identified after careful filtering of sequencing artifacts. A data set is considered to be mutationally saturated when multiple substitutions are dominating. Substitution saturation decreases phylogenetic information, and a highly saturated data set will produce an incorrect phylogeny^20^. To assess the impact of substitution saturation, we randomly selected 100 nuclear orthologs and performed substitution saturation tests. Results showed that the third codon sites of most nuclear orthologs have undergone substantial substitution saturation (Supplementary Table S2), we thus only used the first and second codon sites of the 4325 nuclear orthologs (Supplementary Dataset S2) for subsequent phylogenetic analysis with the concatenation method^35^. Based on the 1854774-nt concatenated alignment of nuclear orthologs from the four species with transcriptome data and the red flour beetle with reference genome data, we used both Maximum Likelihood (ML) and Bayesian Inference (BI) approaches to reconstruct phylogenetic trees. Both maximum likelihood bootstrap values and Bayesian inference posterior probability values (shown as percentages) were 100% at all nodes of the phylogenetic tree (Fig. 2). Specifically, both species from Luciolinae were first clustered into a sister clade, which next grouped with the single species from Lampyrinae (Fig. 2). All the three firefly beetles (Lampyridae) formed a monophyletic group, and the single species from Rhagophthalmidae appeared to locate outside of Lampyridae (Fig. 2).

To reduce the influence of gene tree heterogeneity, we also undertook phylogenetic analysis with the coalescent model using the MP-EST method^36^. Based on the alignment of each orthologous gene with all three codon positions, the ML gene trees with 100 bootstrap replicates were respectively reconstructed by PhyML version 3.0^37^ using the best-fit models generated by jModelTest version 2.1.4^38^. Because poorly supported individual gene trees could reduce phylogenetic signals in inferring species tree^39, 40^, only 2,555 genes with average bootstrap support values greater than 70% were used for MP-EST tree reconstruction. Results showed that the tree topology and bootstrap values of the MP-EST tree using the coalescent method were identical to those of the ML and BI trees reconstructed using the concatenation method (Fig. 2).

For comparison, we additionally reconstructed a phylogeny using a bioluminescence related gene. Because bioluminescence in beetles is produced by catalyzing the oxidation of luciferin by luciferase^41^, the luciferase gene is preferable for this analysis. We used the luciferase gene of *Lampyris noctiluca* (Genbank accession: X89479) as the query to search against the assembled transcripts of each transcriptome using TBLASTN. The best hit from each transcriptome was selected as a candidate, and candidate luciferase genes of the four beetles were searched against NR (NCBI﻿ non-redundant proteins) to ensure accuracy in gene identification. Taking the luciferase gene of *Tribolium castaneum* as the outgroup, we generated an alignment using the five luciferase genes, and phylogenetic trees were built using similar approaches as described elsewhere^19, 42–47^. The resulting tree topologies (Supplementary Fig. S1) were identical to those inferred from 4325 genes (Fig. 2).

### Phylogenetic analysis based on mitochondrial gene sequences

**Figure 3:**
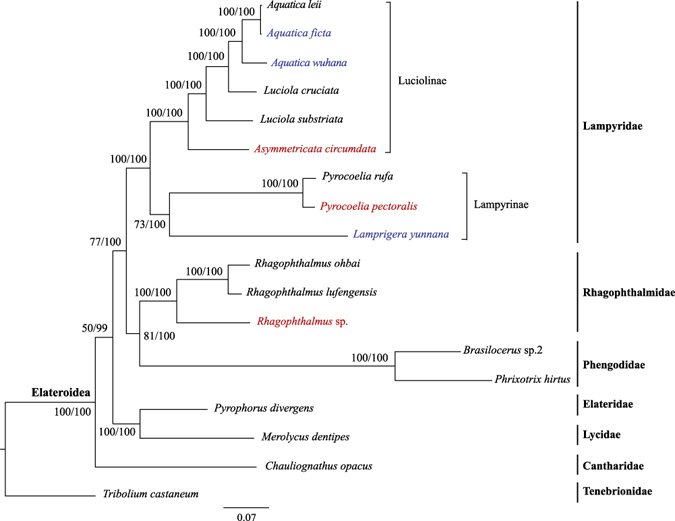
Phylogenetic relationships between Rhagophthalmidae and its related beetle families inferred from the concatenated 13 mitochondrial protein coding genes with the third codon positions removed. Numbers at nodes are the ML bootstrap values/Bayesian posterior probabilities, shown as percentages. Species shown in blue have mitochondrial genes generated from Sanger sequencing, while species in red have mitochondrial genes identified from Illumina-sequenced transcriptomes.

Phylogenetic analysis was also conducted with mitochondrial gene sequences. Firstly, we retrieved published mitogenomes from 12 species of related beetles: *Rhagophthalmus ohbai* (Rhagophthalmidae), *Rhagophthalmus lufengensis* (Rhagophthalmidae), *Pyrocoelia rufa* (Lampyridae), *Luciola cruciate* (Lampyridae), *Luciola substriata* (Lampyridae), *Aquatica leii* (Lampyridae), *Brasilocerus* sp.2 (Phengodidae), *Phrixotrix hirtus* (Phengodidae), *Pyrophorus divergens* (Elateridae), *Merolycus dentipes* (Lycidae), *Chauliognathus opacus* (Cantharidae), and *Tribolium castaneum* (Tenebrionidae) (Fig. 3). Secondly, we newly determined mitogenomes with the traditional Sanger sequencing from three firefly beetles: *A*. *ficta* (Lampyridae), *A*. *wuhana* (Lampyridae) and *L*. *yunnana* (Lampyridae) (Table 3). Thirdly, we identified mitochondrial gene sequences from our newly dertermined transcriptome assemblies of four beetles: *Rhagophthalmus* sp. (Rhagophthalmidae), *A*. *ficta* (Lampyridae), *A*. *circumdata* (Lampyridae), and *P*. *pectoralis* (Lampyridae) (Supplementary Table S1). The firefly *A*. *ficta* has sequence data from both Sanger and Illumina sequencing, but the transcriptome-derived mitogenome sequence is incomplete (Supplementary Table S1), we thus selected the Sanger-based mitogenome sequence (Fig. 3 and Table 3) for further analysis. In total, our dataset of mitogenomes contained 18 beetles. In addition to the outgroup species *T*. *castaneum*, six families of beetles were included: three rhagophthalmids, nine lampyrids, two phengodids, one elaterid, one lycid, and one cantharid (Fig. 3).

The 13 mitochondrial PCGs from each of the 18 beetle species were aligned and concatenated. Substitution saturation analysis revealed that the third codon sites of all examined mitochondrial PCGs have experienced substantial substitution saturation, while the first and second codon sites have not (Supplementary Table S3), we thus just used the first and second codon sites of the 13 mitochondrial genes for subsequent phylogenetic analysis with the concatenation method (Supplementary Dataset S3). The concatenated alignment of the 13 mitochondrial PCGs is 7382 nt in length. Both ML and BI phylogenetic analyses recovered identical phylogenetic trees (Fig. 3). Our trees showed that *Rhagophthalmus* sp. clustered with two other rhagophthalmids with published mitogenomes, and the three rhagophthalmids formed a monophyletic group with a strong support of 100% in both ML and BI analyses (Fig. 3), confirming that our sampled *Rhagophthalmus* beetle indeed belongs to Rhagophthalmidae phylogenetically. As depicted from the trees, Rhagophthalmidae was first allied with Phengodidae as sister groups, and Rhagophthalmidae and Phengodidae formed a monophyletic group with Lampyridae (Fig. 3), confirming that Rhagophthalmidae is not a subgroup within Lampyridae. The long branches in Phengodidae species would not affect our phylogenetic analysis, because we recovered similar trees after removing the two phengodids (Supplementary Fig. S2). We did not perform the coalescent method in the phylogenetic analysis based on mitogenomes, because mitochondrial genes exhibit limited incongruence and the impact of mitochondrial gene tree heterogeneity has been believed to be minimal^48^.

## Discussion

In this study, we present the first whole transcriptome shotgun sequencing data in Rhagophthalmidae and Lampyridae species using massive parallel mRNA sequencing (RNA-seq), providing valuable genome resources for studying the evolution of intriguing traits in these beetles such as bioluminescence. We also newly determined two complete and one nearly complete mitogenome sequences in Lampyridae species. Using various phylogenetic approaches, our transcriptome and mitogenome data unambiguously demonstrate Rhagophthalmidae being distinct from Lampyridae and reject previous hypotheses supporting Rhagophthalmidae as a subgroup within Lampyridae. Our molecular phylogenetic study supports the familial status of Rhagophthalmidae.

The phylogenetic position of Rhagophthalmidae was controversial due to conflicting results inferred from molecular and morphological data. For example, Rhagophthalmidae had been assigned to the family Phengodidae inferred from morphological data^11^; Rhagophthalmidae had been considered to be a subfamily or genus in the family Lampyridae based on morphological, embryological and molecular evidence^8, 12, 14, 15^. However, the overwhelming molecular evidence has consistently recognized the distinctiveness of Rhagophthalmidae, which appeared to be different from both Lampyridae and Phengodidae^7, 9, 13, 17, 22–24, 26, 49, 50^. As a result, the familial status of Rhagophthalmidae has been well established. In this study, we used 4325 nuclear genes derived from transcriptome data and all 13 mitochondrial protein coding genes to conduct phylogenetic inference. Our analyses based on various analytical approaches and several datasets consistently recognized the distinctiveness of Rhagophthalmidae (Figs 2 and 3). Although our study did not involve multiple individuals or populations from each species, intraspecific genetic variation should not impact the phylogenetic resolution at the family or subfamily level. Although our samples are limited due to the lack of genetic material, we included taxa that may represent basal lineages. More importantly, our phylogenetic resolution of Rhagophthalmidae remained consistent after using different analytical approaches and multiple data sets (Figs 2 and 3). In support of our findings, morphological evidence has identified the distinctiveness of Rhagophthalmidae from Lampyridae and Phengodidae^10, 16^, and molecular evidence has revealed that Rhagophthalmidae is a sister group to Phengodidae^9, 13^. In contrast to our findings, two molecular studies argued that Rhagophthalmidae should be placed within Lampyridae^8, 15^. However, this argument was inferred from a short *16S rRNA* gene segment with a length of 506-nt^8, 15^ and cannot be supported by multi-locus data sets^9, 13^. Although broad sampling of taxa is important in molecular phylogenetic studies^8, 15^, sufficient number of gene loci is also required to build a reliable phylogenetic tree^39, 51^. According to our mitogenome analysis, the three luminescent beetle families (Rhagophthalmidae, Lampyridae, and Phengodidae) formed a monophyletic group (Fig. 3), suggesting a single origin of bioluminescence in the common ancestor of these beetles. This finding is consistent with a recent phylogenetic analysis based on mitogenomes^9^ but inconsistent with another study based on a combination of mitochondrial and nuclear genes^13^. The contrasting results between mitochondrial and nuclear genes call for an in-depth study by adding more taxa and more genome-scale data, which will help reconstruct a reliable phylogeny and make a conclusive inference on the evolution of bioluminescence in beetles.

**Figure 4:**
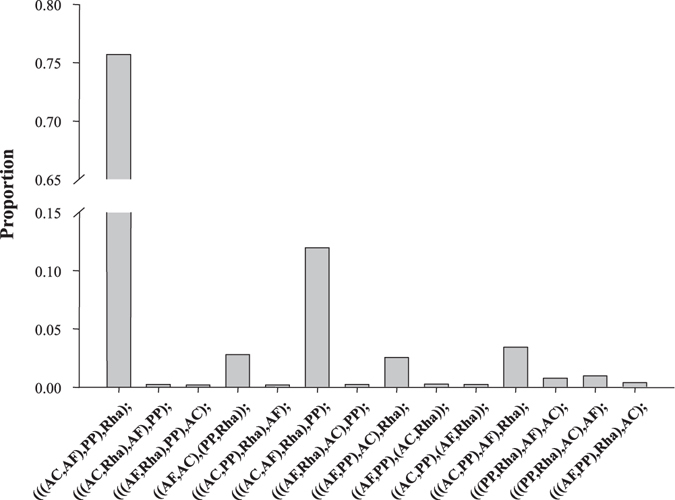
A histogram depicting the proportions of 14 tree topologies with average bootstrap values above 70%, inferred from 2555 orthologous genes in the MP-EST analysis. Rha, *Rhagophthalmus* sp.; AC, *Asymmetricata circumdata*; AF, *Aquatica ficta*; PP, *Pyrocoelia pectoralis*.

Phylogenomics refers to phylogenetic analysis involving genome-scale data. Phylogenomics has been believed to outcompete single-gene phylogenetics, which frequently yielded conflicting results caused by stochastic errors from small-scale data sets^20, 51^. However, systematic errors are still present after adding more data^52^. There are three major challenges that could generate strong incongruence in phylogenomic analysis: substitution saturation, nucleotide compositional bias, and different tree reconstruction methods^20^. We took the following steps to overcome these challenges. First, we undertook substitution saturation tests and removed the third codon positions of all genes that may have undergone substantial substitution saturation. Both nuclear and mitochondrial gene data sets with the first and second codon positions consistently supported Rhagophthalmidae to be an independent group that is distinct from Lampyridae (Figs 2 and 3). Second, we used the deduced protein sequences to reduce nucleotide compositional bias. Our result showed that the BI tree topologies inferred from protein sequences (Supplementary Fig. S3) were identical to those inferred from nucleotide sequences (Figs 2 and 3), suggesting that nucleotide compositional bias is not a major factor affecting our phylogenetic analysis. Third, we used both ML and BI methods with nuclear and mitochondrial gene data sets, and identified no incongruence between the two tree reconstruction methods (Figs 2 and 3). In addition, given the prevalence of gene tree heterogeneity^53–55^, we also conducted phylogenetic analysis with the coalescent method, which has been proved to generate accurate and congruent phylogenies in the presence of heterogeneous gene trees^56, 57^. The coalescent method implemented in the MP-EST program^36^ was applied to our transcriptome-derived nuclear gene data set of 2555 putative orthologous genes, and yielded a phylogenetic tree identical to the concatenation-based ML and BI trees (Fig. 2). Indeed, after examining the proportions of 14 possible topologies inferred from the 2555 orthologous genes, we found that most genes (75.7%) supported the topology shown in Fig. 2 and the average proportion of other 13 topologies is 1.9% (Fig. 4 and Supplementary Table S4), suggesting a low level of gene tree heterogeneity among beetles studied in this work. This study proved usefulness of our genome-scale data in phylogenetic analysis, and will help to illuminate the origin and evolution of bioluminescence in Lampyridae and other luminescent beetles.

## Materials and Methods

### Ethics statement

All beetle species used in this study were sampled in the field. No specific permits were required, and no endangered or protected species were involved. All experiments on the beetles were conformed to the rules and guidelines on animal experimentation in China.

### Taxon sampling and DNA extraction

Live larvae or adults of beetles studied here were sampled at five locations in China (Supplementary Table S5). All these individuals were stored at −80 °C after freezing in liquid nitrogen. For mitogenome sequencing, genomic DNAs of the three species (*A*. *ficta*, *A*. *wuhana* and *L*. *yunnana*) were isolated from thoracic muscles of adult individuals with the Qiagen DNeasy kits.

### Whole-body transcriptome sequencing (RNA-seq)

Following the manufacturer’s protocol, total RNAs were isolated using Trizol (Invitrogen) from whole bodies of four individuals: one adult individual of *Rhagophthalmus* sp. and three larva individuals of firefly beetles (*A*. *ficta*, *A*. *circumdata*, and *P*. *pectoralis*). Four paired-end libraries with an insert size of approximately 200 bp were constructed using the Illumina Truseq RNA sample prep kit according to the manufacturer’s protocol. Details of constructing RNA-seq libraries were previously described^19^. All libraries were sequenced commercially to generate paired-end reads of average length 101-bp on the Illumina HiSeq 2000 sequencing platform^58^.

### De novo assembly and unigene annotation

Sequence quality of each RNA-seq sample was assessed by FastQC version 10.1 (www.bioinformatics.bbsrc.ac.uk/projects/fastqc). Trimmomatic version 0.32^59^ was used to trim out low quality sequences, ambiguous sequences and artificial sequences such as residual adaptors and Illumina specific sequences as described elsewhere^60^. Four de novo transcriptome assemblies were constructed by the Trinity program^61^ with default settings. Because de novo transcriptome assemblies involve many contigs in a contig cluster, we required a minimum expression filter of one fragment per kilobase of exon per million fragments mapped (FPKM) and filtered the contigs with FPKM value smaller than one; the contig with the highest expression level in a contig cluster was selected to be a unigene for downstream analyses^60^. The resulting unigenes were used for BLASTX searches^27^ and unigene annotations based on the NR, Swiss-prot and COG databases, with an E-value cutoff of 1e-5. The GO annotation was conducted by Blast2GO software^62^ with default parameters using the NR blast results in XML format.

### PCR amplifications and mitogenomes annotation

To amplify the three mitogenomes (*A*.*ficta*, *A*.*wuhana* and *L*.*yunnana*), dozens of primer pairs were designed according to published firefly mitogenome sequences^32–34^. Details of PCR amplification and sequencing procedure were described in our previous study^33^. We defined the 13 PCGs of the three species by multiple sequence alignments with related species using MEGA version 5.20^63^. The tRNAs were identified by tRNAscan-SE^64^. The published mitogenomes used here were downloaded from the GenBank database (http://www.ncbi.nlm.nih.gov/genbank) under accession numbers as follows: *Tribolium castaneum*, NC_003081; *Brasilocerus* sp.2., KJ938490; *Pyrophorus divergens*, NC_009964; *Chauliognathus opacus*, NC_013576; *Rhagophthalmus ohbai*, NC_010964; *Rhagophthalmus lufengensis*, NC_010969; *Pyrocoelia rufa*, NC_003970; *Luciola cruciata*, NC_022472; *Luciola substriata*, NC_027176; *Aquatica leii*, NC_025276; *Phrixotrix hirtus*, KM923891; *Merolycus dentipes*, HQ232815.

### Ortholog identification and phylogenomic analysis

The cDNA sequences of the red flour beetle (*Tribolium castaneum* 3.0 Assembly) were downloaded from BeetleBase^65^ (http://www.Beetlebase.org), and compared with the transcriptome-derived unigenes by reciprocal (or bi-directional) TBLASTX searches^27^. We identified putative orthologs by examining reciprocal best blast hits. We discarded putative orthologs with a BLAST score ratio of the second best-hit to the first best-hit greater than 0.8, which can exclude potential paralogs in our phylogenetic analysis. All putative orthologs were aligned by PRANK version 100802^28^, and poorly aligned positions and divergent regions were removed by GBLOCKS version 0.91b^29^. In addition, the alignments with an aligned region shorter than 100-nt were discarded.

All the 13 mitochondrial PCGs and 100 randomly selected nuclear orthologs were used to examine the substitution saturation by DAMBE^66^. According to the Akaike information criterion (AIC) and Bayesian information criterion (BIC)^67^, we ran the jModelTest version 2.1.4 program^38^ separately to select the best-fit models of nucleotide substitution for concatenated nuclear and mitochondrial gene alignments after removal of the saturated third codon positions. For the concatenated nuclear genes, RAxML version 7.2.6^68^ was used to reconstruct the ML tree under the GTR+GAMMA model with 100 bootstrap replicates, and MrBayes version 3.2.6^69^ was applied to reconstruct the BI tree using the recommended GTR+I+G model with 0.5 million generations^45, 47^. To reduce the impact of gene tree heterogeneity, we also undertook phylogenetic analysis with the coalescent model using the MP-EST method^36^. For the concatenated 13 mitochondrial PCGs, the ML tree was reconstructed by RAxML version 7.2.6 with 1000 bootstrap replicates under recommended GTR+GAMMA model, and the concatenated sequence was partitioned by different genes to estimate and optimize individual α-shape parameters, GTR-rates, and base frequencies for each gene. MrBayes version 3.2.6 was used to reconstruct the BI tree with one million generations and the concatenated sequence was partitioned according to different models: HKY+G model for *ND4L*, GTR+G model for *ND1* and *ATP6*, and GTR+I+G model for the other 10 mitochondrial genes.

In addition, we used deduced protein sequences in our phylogenomic analysis, aiming to reduce the impact of nucleotide compositional bias. Briefly, protein sequences of the nuclear and mitochondrial genes were deduced and aligned by MEGA version 5.20^63^. ProtTest version 3.41^70^ was applied to select the best-fit model of protein sequence evolution following the Akaike information criterion (AIC). The LG+I+G model was determined to provide the best fit to the concatenated protein sequences of nuclear genes, while the MtRev+I+G model was selected for the concatenated protein sequences of mitochondrial genes. Phylogenetic trees were reconstructed by MrBayes version 3.2.6^69^ with as many generations as required.

## Electronic supplementary material

Supplementary Information

Dataset S1

Dataset S2

Dataset S3
