# Supplementary material for: Transcriptome sequencing and phylogenetic analysis of four species of luminescent beetles
Source: Sci Rep. 2017 May 12;7:1814. doi: 10.1038/s41598-017-01835-9 (PMC5431921; doi:10.1038/s41598-017-01835-9)
Supplement: Supplementary file 1 — Supplementary Information [file 41598_2017_1835_MOESM1_ESM.pdf]

Supplementary Information for

Transcriptome sequencing and phylogenetic analysis of four species of luminescent beetles

Kai Wang, Wei Hong, Hengwu Jiao, and Huabin Zhao* (huabinzhao@whu.edu.cn)

This document includes:

Fig. S1 – Fig. S3

Table S1 – Table S5.

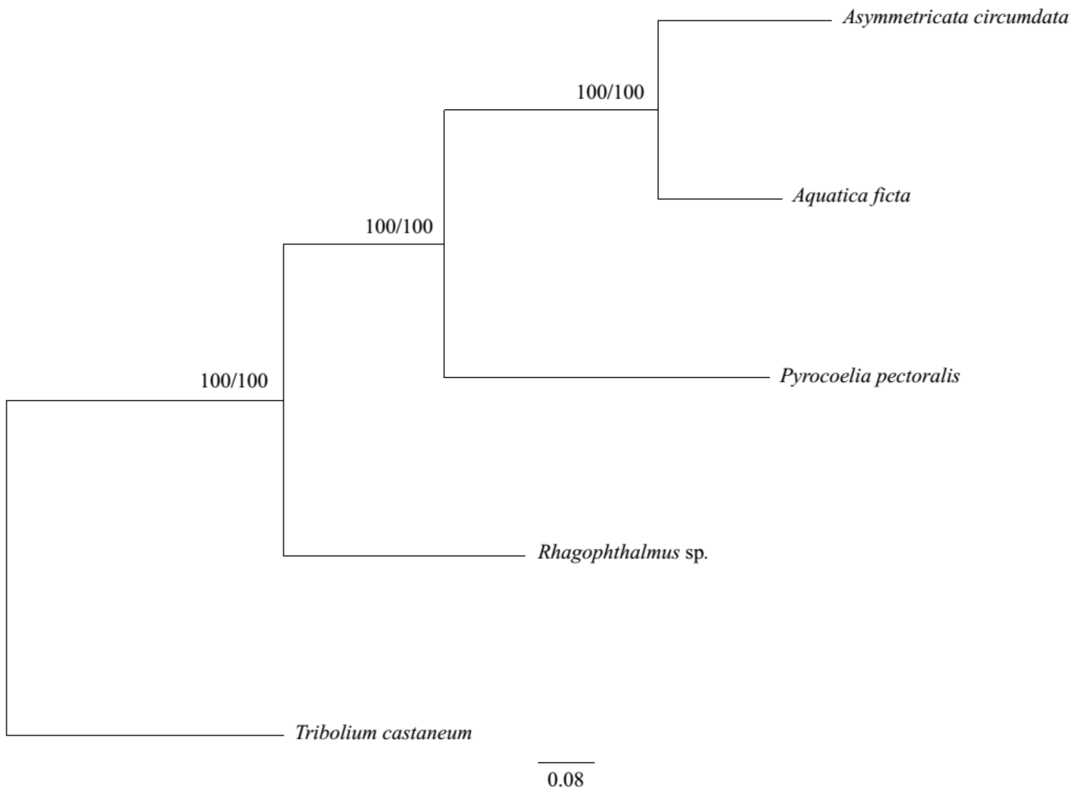

Figure S1. Phylogenetic trees inferred from the luciferase genes. Numbers at nodes are the ML bootstrap values/Bayesian posterior probabilities shown as percentages.

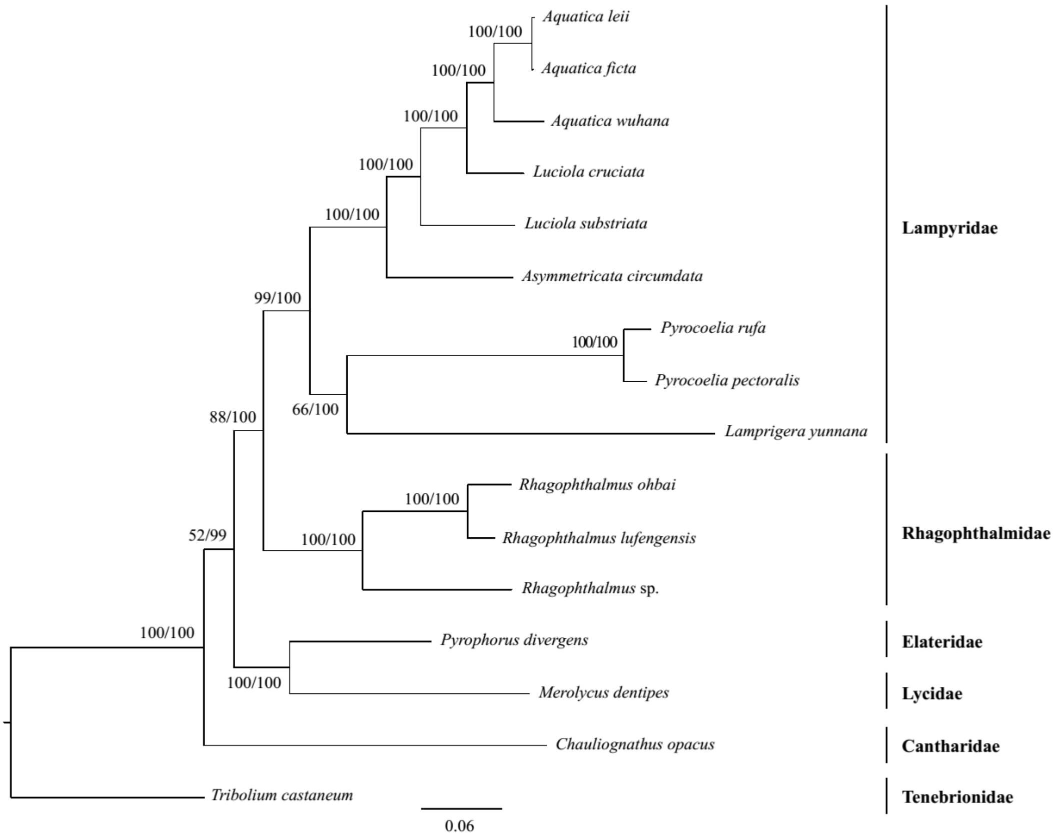

Figure S2. Phylogenetic trees reconstructed from the concatenated 13 mitochondrial protein coding genes, after removing the two phengodids. Numbers at nodes are the ML bootstrap values/Bayesian posterior probabilities, shown as percentages.

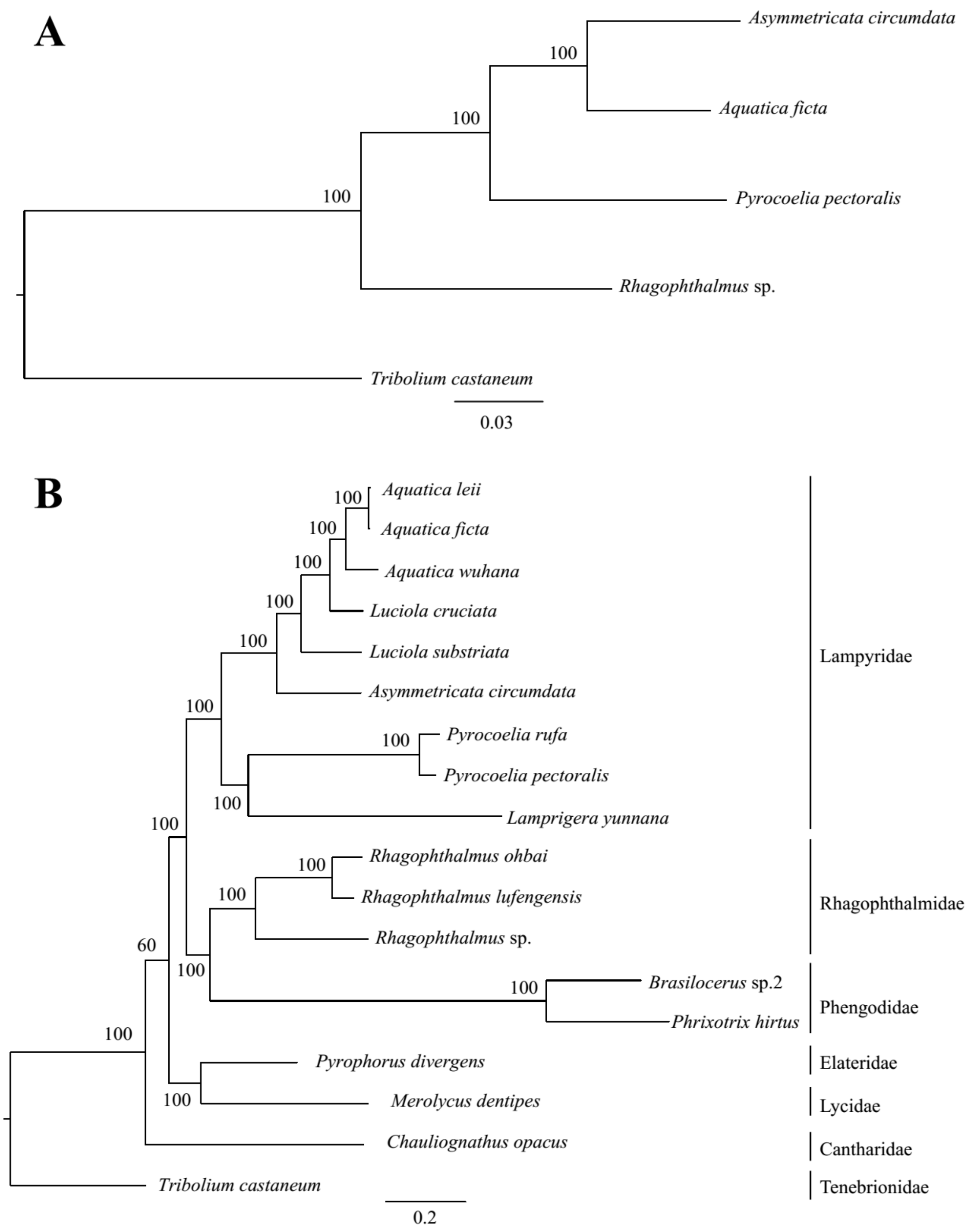

Figure S3. Phylogenetic trees inferred from deduced protein sequences of 4325 nuclear genes **(A)** and 13 mitochondrial protein coding genes **(B)**. Numbers at nodes are the Bayesian posterior probabilities as percentages.

Table S1. Statistics of the 13 mitochondrial protein-coding genes identified from the four transcriptomes. Abbreviation: na, not available (due to either incomplete sequencing or low sequence coverage).

Gene	*Asymmetricata circumdata*		*Aquatica ficta*		*Pyrocoelia pectoralis*		*Rhagophthalmus* sp.	
	**Length (bp)**	**Start/stop codon**	**Length (bp)**	**Start/stop codon**	**Length (bp)**	**Start/stop codon**	**Length (bp)**	**Start/stop codon**
ND2	666	n.a./n.a.	986	n.a./TAG	394	ATA/n.a.	666	ATT/n.a.
COI	1545	ATT/TAA	1540	ATT/T+tRNA	1534	n.a./T+tRNA	1534	n.a./T+tRNA
COII	648	n.a./TAA	679	ATG/T+tRNA	604	n.a./T+tRNA	679	ATG/T+tRNA
ATP8	n.a.	n.a.	141	n.a./TAA	66	n.a./TAA	150	ATT/TAA
ATP6	519	n.a./TAA	675	ATG/TAA	670	ATG/T+tRNA	670	ATG/T+tRNA
COIII	786	ATG/TAA	784	ATG/T+tRNA	784	ATG/T+tRNA	784	ATG/T+tRNA
ND3	231	n.a./n.a.	352	ATT/T+tRNA	352	ATA/T+tRNA	352	ATA/T+tRNA
ND5	1293	n.a./TAA	1340	n.a./n.a.	1550	n.a./n.a.	1389	n.a./n.a.
ND4	1063	n.a./n.a.	1112	n.a./n.a.	1321	ATT/T+tRNA	1117	n.a./n.a.
ND4L	189	n.a./TAA	265	n.a./TAA	285	n.a./TAA	261	n.a./TAA
ND6	486	ATT/TAA	419	n.a./TAA	486	ATA/TAA	489	ATA/TAA
CYTB	1134	ATG/TAA	1132	ATG/T+tRNA	1129	ATG/T+tRNA	1129	ATG/T+tRNA
ND1	933	ATT/TAG	945	ATT/TAG	909	ATA/n.a.	948	ATG/TAG

Table S2. Substitution saturation tests for the 100 selected nuclear orthologs by DAMBE.

Orthologs	Substitution saturation of the 1st and 2nd codon sites	DAMBE result for the 3rd sites			Substitution saturation of the 3rd codon sites
		Iss	Iss.c (AS)	Probability	
ortholog_1.fas	N	0.7122	0.7466	0.3664	Y
ortholog_10.fas	N	0.7110	0.7317	0.5399	Y
ortholog_100.fas	N	0.8450	0.7780	0.1422	Y
ortholog_1000.fas	N	0.7827	0.8163	0.5193	Y
ortholog_1001.fas	N	0.6878	0.7123	0.3777	Y
ortholog_1002.fas	N	0.7600	0.8185	0.2169	Y
ortholog_1003.fas	N	0.8154	0.8638	0.2863	Y
ortholog_1005.fas	N	0.7226	0.7160	0.7746	Y
ortholog_1006.fas	N	0.7422	0.7344	0.8296	Y
ortholog_1007.fas	N	0.6695	0.7243	0.1022	Y
ortholog_1008.fas	N	0.6862	0.8058	0.0066	N
ortholog_1009.fas	N	0.7037	0.7808	0.0678	Y
ortholog_1011.fas	N	0.8328	0.7527	0.033	Y
ortholog_1012.fas	N	0.7372	0.7181	0.3873	Y
ortholog_1013.fas	N	0.7372	0.7181	0.3873	Y
ortholog_1014.fas	N	0.7707	0.7172	0.0571	Y
ortholog_1015.fas	N	0.7863	0.7452	0.2092	Y
ortholog_1016.fas	N	0.7264	0.7151	0.7279	Y
ortholog_1017.fas	N	0.7073	0.7452	0.2933	Y
ortholog_1018.fas	N	0.7910	0.7947	0.9334	Y
ortholog_1019.fas	N	0.6748	0.7125	0.1407	Y
ortholog_102.fas	N	0.7970	0.7219	0.0159	Y
ortholog_1020.fas	N	0.7645	0.7866	0.5782	Y
ortholog_1021.fas	N	0.7630	0.7156	0.1273	Y
ortholog_1022.fas	N	0.7800	0.7139	0.0286	Y
ortholog_1023.fas	N	0.6532	1.0493	0	N
ortholog_1024.fas	N	0.7625	0.7142	0.1078	Y
ortholog_1025.fas	N	0.7935	0.7598	0.3677	Y
ortholog_1026.fas	N	0.5867	0.7118	0	N
ortholog_1027.fas	N	0.7416	0.7156	0.256	Y
ortholog_1028.fas	N	0.6992	0.7794	0.0459	N
ortholog_1029.fas	N	0.8247	0.7459	0.0299	Y
ortholog_103.fas	N	0.7894	0.7255	0.051	Y
ortholog_1030.fas	N	0.8312	0.8185	0.7894	Y
ortholog_104.fas	N	0.6740	0.7166	0.0732	Y
ortholog_106.fas	N	0.7869	0.7851	0.487	Y
ortholog_107.fas	N	0.7731	0.7495	0.5296	Y
ortholog_108.fas	N	0.7419	0.7598	0.6712	Y
ortholog_109.fas	N	0.7555	0.7129	0.1185	Y
ortholog_11.fas	N	0.7904	0.7135	0.0039	Y
ortholog_111.fas	N	0.6102	0.8078	0.0001	N
ortholog_112.fas	N	0.7130	0.7121	0.9736	Y
ortholog_113.fas	N	0.7181	0.7354	0.6073	Y
ortholog_114.fas	N	0.7336	0.8281	0.0505	Y
ortholog_115.fas	N	0.7064	0.7125	0.8292	Y
ortholog_116.fas	N	0.7478	0.7195	0.4031	Y

ortholog_117.fas	N	0.6451	0.7344	0.0175	N
ortholog_118.fas	N	0.6835	0.7245	0.0487	N
ortholog_12.fas	N	0.8454	0.7947	0.2569	Y
ortholog_120.fas	N	0.6981	0.7659	0.0931	Y
ortholog_121.fas	N	0.7080	0.7137	0.844	Y
ortholog_123.fas	N	0.6872	0.7137	0.37	Y
ortholog_124.fas	N	0.7120	0.7143	0.918	Y
ortholog_125.fas	N	0.8369	0.8710	0.0994	Y
ortholog_126.fas	N	0.6684	0.7310	0.0016	N
ortholog_127.fas	N	0.7986	0.7127	0.0012	N
ortholog_128.fas	N	0.7037	0.7161	0.7023	Y
ortholog_13.fas	N	0.6918	0.7369	0.2765	Y
ortholog_130.fas	N	0.7915	0.7914	0.9974	Y
ortholog_131.fas	N	0.7534	0.7297	0.4882	Y
ortholog_132.fas	N	0.7586	0.7219	0.7346	Y
ortholog_133.fas	N	0.7166	0.7187	0.9506	Y
ortholog_134.fas	N	0.7013	0.7118	0.6861	Y
ortholog_136.fas	N	0.6835	0.7195	0.2938	Y
ortholog_137.fas	N	0.6971	0.7729	0.1144	Y
ortholog_138.fas	N	0.6905	0.7965	0.0154	N
ortholog_139.fas	N	0.7536	0.8748	0.0225	N
ortholog_14.fas	N	0.7714	0.8998	0.0231	Y
ortholog_140.fas	N	0.7698	0.7420	0.4472	Y
ortholog_141.fas	N	0.7463	0.7158	0.303	Y
ortholog_142.fas	N	0.6953	0.8710	0.0007	N
ortholog_143.fas	N	0.8873	0.7402	0	Y
ortholog_144.fas	N	0.7708	0.7148	0.0706	Y
ortholog_145.fas	N	0.7432	0.7164	0.2188	Y
ortholog_146.fas	N	0.7116	0.9299	0.0001	N
ortholog_147.fas	N	0.6724	0.7882	0.0122	N
ortholog_148.fas	N	0.8348	1.0702	0.0024	N
ortholog_150.fas	N	0.7467	0.9473	0.0005	N
ortholog_151.fas	N	0.7058	0.7180	0.7156	Y
ortholog_152.fas	N	0.7157	0.7127	0.9201	Y
ortholog_153.fas	N	0.7250	0.7385	0.7172	Y
ortholog_154.fas	N	0.7463	0.7354	0.7691	Y
ortholog_155.fas	N	0.7390	0.7271	0.5376	Y
ortholog_156.fas	N	0.6537	0.7118	0.0417	N
ortholog_158.fas	N	0.7519	0.7511	0.9805	Y
ortholog_159.fas	N	0.7529	0.7983	0.3759	Y
ortholog_16.fas	N	0.7761	0.8185	0.3611	Y
ortholog_162.fas	N	0.7819	0.7511	0.4011	Y
ortholog_163.fas	N	0.6845	0.7181	0.1213	Y
ortholog_164.fas	N	0.7958	0.7278	0.0266	Y
ortholog_165.fas	N	0.7129	0.9192	0.0001	N
ortholog_167.fas	N	0.7646	0.7129	0.0604	Y
ortholog_168.fas	N	0.7588	0.7130	0.0426	Y
ortholog_169.fas	N	0.6946	0.7223	0.2113	Y
ortholog_17.fas	N	0.7345	0.7309	0.9255	Y
ortholog_171.fas	N	0.6105	0.7205	0.0007	N
ortholog_173.fas	N	0.7531	0.7535	0.9914	Y

ortholog_174.fas	N	0.7637	0.7930	0.5271	Y
ortholog_175.fas	N	0.7492	0.7511	0.9596	Y
ortholog_176.fas	N	0.7140	0.7222	0.7866	Y

Table S3. Substitution saturation analysis of mitochondrial genes by DAMBE.

Abbreviations: N, no; Y, yes.

Gene	Substitution saturation of the 1st and 2nd codon sites	DAMBE result for the 3rd sites			Substitution saturation of the 3rd codon site
		Iss	Iss.c(S)	Probability	
ATP6	N	0.7729	0.6087	0.0000	Y
COX1	N	0.6869	0.7143	0.1490	Y
COX2	N	0.7436	0.6259	0.0001	Y
COX3	N	0.7053	0.6482	0.0351	Y
CYTB	N	0.7060	0.6838	0.2703	Y
ND1	N	0.7031	0.6646	0.1471	Y
ND2	N	0.7154	0.5035	0.0001	Y
ND3	N	0.7526	0.4780	0.0000	Y
ND4	N	0.7423	0.6795	0.0110	Y
ND4L	N	0.7305	0.5029	0.0002	Y
ND5	N	0.7325	0.6915	0.0655	Y
ND6	N	0.7918	0.5714	0.0000	Y

Table S4. The distribution of 14 gene tree topologies inferred from the 2555 orthologs with average bootstrap values above 70% in the MP-EST analysis. Rha, *Rhagophthalmus* sp.; AC, *Asymmetricata circumdata*; AF, *Aquatica ficta*; PP, *Pyrocoelia pectoralis*.

Topology	Counts	Percentage (%)
(((AC, AF), PP), Rha)	1934	75.695%
(((AC, Rha), AF), PP)	6	0.235%
(((AF, Rha), PP), AC)	5	0.196%
((AF, AC), (PP, Rha))	72	2.818%
(((AC, PP), Rha), AF)	5	0.196%
(((AC, AF), Rha), PP)	306	11.977%
(((AF, Rha), AC), PP)	6	0.235%
(((AF, PP), AC), Rha)	65	2.544%
((AF, PP), (AC, Rha))	7	0.274%
((AC, PP), (AF, Rha))	6	0.235%
(((AC, PP), AF), Rha)	88	3.444%
(((PP, Rha), AF), AC)	20	0.783%
(((PP, Rha), AC), AF)	25	0.978%
(((AF, PP), Rha), AC)	10	0.391%
Total	2555	100%

Table S5. Taxa studied and sampling sites in China.

Family	Subfamily	Species	Province	Collection site
**Lampyridae**	**Lampyrinae**	*Pyrocoelia pectoralis*	Hubei	Ezhou City
		Lampyrigera yunnana	Sichuan	Leshan City
	**Luciolinae**	*Aquatica ficta*	Hubei	Xianning City
		Aquatica wuhana	Hubei	Wuhan City
		Asymmetricata circumdata	Hainan	Danzhou City
**Rhagophthalmidae**		*Rhagophthalmus* sp.	Hainan	Danzhou City
